# One-Pot Hydrothermal Synthesis of La-Doped ZnIn_2_S_4_ Microspheres with Improved Visible-Light Photocatalytic Performance

**DOI:** 10.3390/nano10102026

**Published:** 2020-10-14

**Authors:** Tiekun Jia, Ming Liu, Chunyang Zheng, Fei Long, Zhiyu Min, Fang Fu, Dongsheng Yu, Jili Li, Joong Hee Lee, Nam Hoon Kim

**Affiliations:** 1 School of Materials Science and Engineering, Luoyang Institute of Science and Technology, Luoyang 471023, China; cocoaa99@163.com (C.Z.); fufang1@126.com (F.F.); dongsh_yu@163.com (D.Y.); lijili328@126.com (J.L.); 2 School of Materials Science and Engineering, Guilin University of Technology, Guilin 541004, China; longf@glut.edu.cn; 3 Department of Nano Convergence Engineering, Jeonbuk National University, Jeonju 54896, Korea; jhl@jbnu.ac.kr (J.H.L.); nhk@jbnu.ac.kr (N.H.K.)

**Keywords:** ZnIn_2_S_4_, doping, visible-light, photocatalytic degradation

## Abstract

Impurity element doping is extensively taken as one of the most efficient strategies to regulate the electronic structure as well as the rate of photogenerated charge separation of photocatalysts. Herein, a one-pot hydrothermal synthesis process was exploited to obtain La-doped ZnIn_2_S_4_ microspheres, aiming at gaining insight into the role that doping ions played in the improvement of pollutant photodegradation. Systematical characterization means, comprising of X–ray photoelectron spectroscopy (XPS), ultraviolet–visible (UV–vis) diffuse reflection spectroscopy and Raman spectra, combination with high-resolution transmission electron microscopy (HRTEM), were employed to in depth reveal the concomitancy of La ions and ZnIn_2_S_4_ crystal lattice. The results showed that the La-doped ZnIn_2_S_4_ samples exhibited a slightly wider and stronger spectral absorption than pristine ZnIn_2_S_4_; and the specific surface area of doped ZnIn_2_S_4_ samples was a bit larger. The La-doped ZnIn_2_S_4_ electrodes showed improved photocurrent response, and the photocurrent density reached a maximum value at La content of 1.5 wt%. As expected, La-doped ZnIn_2_S_4_ samples exhibited a remarkable enhancement of photocatalytic behaviour toward the photodegradation of tetracycline hydrochloride (TCH) and methyl orange (MO). The prominently enhanced photoactivity of doped ZnIn_2_S_4_ samples was due to the synergistic effect of the elevated visible-light absorption ability and effective photogenerated charge carriers’ separation.

## 1. Introduction

As we are stepping into 21st century with rapid development of economic and manufacturing, more and more organic pollutants, such as dyes and tetracycline hydrochloride (TCH), from the textile industry or pharmaceutical industry, will be increasingly discharged into waste water. These pollutants have commonly intrinsic characters of highly chemical stability, toxicity, and non-biodegradability, probably giving rise to a serious threat to eco-environment, aquatic breeding, and even health of human beings by poisoning the food chain [1,2]. Therefore, the environmental pollution problem is increasingly becoming more serious than ever, and is likely to emerge as a huge obstacle for the sustainable development of human beings. Photocatalytic technology with notable merits of easy control, degrading pollutants sufficiently and bringing about no secondary pollution, is considered as one of the most appealing solutions to solve the aforementioned challenge [1,2,3,4,5,6]. However, the performance of photocatalysts has almost suffered from a limited window for response to the solar spectrum and poor efficiency of photogenerated charge carrier separation for a long period. Thus, the design and synthesis of novel photocatalyst are critical for achieving enhanced photo-reactivity.

ZnIn_2_S_4_ (ZIS), a typical II-III_2_-VI_4_ ternary chalcogenide, is endowed with the characteristics of excellent light-harvesting capacity and suitable band energy structure because of its relatively narrow band-gap and negative conductive band potential [7]. ZIS has already drawn substantial attention and readily found wide applications in the field of photocatalysis, such as pollutant photodegradation [8,9,10,11], hydrogen evolution from water splitting [12,13,14,15,16,17], and CO_2_ photoreduction [18,19]. Unfortunately, limited to its speedy photoinduced charge recombination and low quantum rate, the photo-reactivity of pristine ZIS is not sufficient for applications. Thus, substantial efforts have been devoted to promote the photocatalytic behavior of ZIS_-_based photocatalysts by heterogeneous coupling [18,19,20,21], noble-metal loading [22,23,24,25,26,27,28], and impurity element doping [29,30,31,32,33]. Among them, impurity element doping is universally taken as one of the most efficient strategies to regulate the electronic structure as well as the rate of photogenerated charge separation of photocatalysts [34,35,36]. Previously, Guo et al. initiated interesting research on transition metal (Cr, Mn, Fe, Co)-doped ZIS photocatalysts for hydrogen evolution, and discussed the effect of different dopant ions on the photocatalytic performance. The results showed that the introduction of Cr, Fe, and Co supressed the photocatalytic behaviour for hydrogen evolution, while the hydrogen evolution rate of a Mo-doped ZIS sample was larger than that of pure ZIS [29]. Tan et al. reported the research about the doping effect of Sm on the adsorption and photocatalytic performance of ZIS photocatalysts toward Rhodamine (RhB) and methyl orange (MO) photodegradation, indicating that the efficiently enhanced absorption ability resulting from Sm doping indeed promoted the photocatalytic degradation rate [31]. Gao et al. reported a simple route for the synthesis of Fe-doped ZIS, and investigated the photocatalytic performance toward the photodegradation of 2,4,6-tribromophenol. The results verified that Fe-doped ZIS photocatalysts were more efficient, stable, and durable in the photodegradation of 2,4,6-tribromophenol than pure ZIS and titania particle size 25 (P25) [32]. Besides the aforementioned work on metal ions, Xie et al. designed and developed O-doped ZIS nanosheets with novel structure, acting as a platform to improve the photocatalytic activity due to the increased density of states and accelerated separation rate of photoexcited carriers [33]. These findings in previous studies are interesting and impressive, thus, they inspire us to pursue for other effective doping in ZIS photocatalyst with improved photo-reactivity toward organic pollutant degradation.

For a lanthanide atom, its 4f orbital is not fully occupied and 5d orbital is empty, which can afford efficient electron capture. Such a feature is beneficial to reduce the recombination rate of photoexcited charge carriers [37]. As a representative of lanthanide group, La ion with a stable valence state (3+) can augment the quantum yield by extending the lifetime of photoexcited carriers due to the shallow potential trap induced by its full electronic configuration [38]. Accordingly, La doping is widely utilized as an effective approach to modulate the electronic structure and catalytic activity of photocatalysts [37,38,39,40,41]. We previously adopted a hydrothermal process to prepare La-doped ZnO nanowires. The introduction of La^3+^ changed the band energy structure, and decreased the diameter of nanowires. The photocatalytic performance of La-doped ZnO nanowires was obviously improved correspondingly [38]. Recent studies about La-doped BiOCl and La-doped (BiO_2_)_2_CO_3_ photocatalysts proved that La doping indeed promoted the charge carriers separation and migration efficiency [39,40]. Unfortunately, there are limited reports on the synthesis of La-doped ZIS and pollutant photo-degradation toward MO and tetracycline hydrochloride (TCH). Enlightened by the aforementioned studies, we conceived a one-pot hydrothermal synthesis process to fabricate La-ZIS microspheres. We planned to choose MO and TCH as target pollutants to investigate the photocatalytic behavior of the as-prepared samples. It is anticipated that the modification approach by doping La ion into ZIS will make a great contribution to the photocatalytic reactivity of ZIS based photocatalysts.

Herein, a one-pot hydrothermal synthesis process was developed to obtain La-doped ZnIn_2_S_4_ microspheres, and systematical characterization means, comprising X–ray photoelectron spectroscopy (XPS), ultraviolet–visible (UV–vis) diffuse reflection spectroscopy and Raman spectra, in combination with high-resolution transmission electron microscopy (HRTEM), which were employed to completely characterize the as-prepared samples. To evaluate the effect of La doping on the catalytic activity, we carried out photodegradation experiments of MO and TCH over the as-prepared ZIS based samples. The results indicated that La-doped ZIS samples exhibited a remarkable enhancement of photocatalytic behavior. As far as we know, this study is the first report about the synthesis and improved photocatalytic performance of La-doped ZIS.

## 2. Experimental Details

### 2.1. Materials

All the chemicals, containing indium trichloride tetrahydrate (InCl_3_·4H_2_O), zinc dichloride (ZnCl_2_), thioacetamide (TAA), lanthanum nitrate pentahydrate (La(NO_3_)_3_·5H_2_O), TCH, and methyl orange (MO) were analytical grade, and were purchased from Sinopharm Chemical Reagent CO., Ltd., Shanghai, China.

### 2.2. Synthesis of Pure ZnIn_2_S_4_ (ZIS) and La-Doped ZIS

Pure ZIS (P-ZIS) and La-doped ZIS samples were obtained via a one-pot hydrothermal synthesis process as follows. For the P-ZIS sample, 1 mmol of ZnCl_2_, and 2 mmol of InCl_3_·4H_2_O were added into 80 mL deionized water (DI.W) with vigorous stirring. After a stirring time of 20 min, 8 mmol of TAA was added into the above solution. Subsequently, the mixed solution was stirred for another 40 min at room temperature. After that, it was put into a Teflon-lined autoclave (100 mL), which would be heated to 160 °C, and held for 15 h. After being cooled to room temperature, the precipitate was treated by centrifuging and washing with DI.W and alcohol repeatedly. The resulting yellow powders were finally achieved after drying at 60 °C for 10 h. For La-doped ZIS, a given amount of La(NO_3_)_3_·5H_2_O was also added into the above reaction system. By varying the amount of La(NO_3_)_3_·5H_2_O, a series of La-doped ZIS samples with La different molar ratio of 0.5 at%, 1 at%, 1.5 at%, and 2 at%, was obtained and respectively denoted as 0.5L-ZIS, 1L-ZIS, 1.5L-ZIS and 2L-ZIS for convenience.

### 2.3. Characterization

The crystalline structure of P-ZIS and doped ZIS samples was analyzed on an X–ray diffractometer (D8 Advance, Bruker, Billerica, MA, USA) equipped with a copper source. Morphology observation and micro-structure investigation were conducted on a field-emission electron scanning microscope (Hitachi S-4800, Tokyo, Japan) combined with a transmission electron microscope (JEM-2100F, JEOL Ltd., Tokyo, Japan). The investigation of surface components and chemical status was realized by the utilization of the X-ray photoelectron spectrometer (XPS, ESCALAB 250Xi, Thermo Fisher Scientific corporation, New York, NY, USA), and the 284.6 eV of C1s was taken as the criterion to correct binding energies of other elements. The determination of the specific surface area was accomplished by using the method of nitrogen adsorption and desorption on a sorption analyzer (NOVA 2000e, Boynton Beach, FL, USA). Raman spectra were collected on a Renishaw invia spectrometer (Renishaw, London, UK) by the aid of a 514 nm laser line (Ar^+^) as excitation source. Assigning barium sulphate as the reference sample, UV–vis diffuse reflectance spectra of P-ZIS and doped ZIS were collected on a UV–vis spectrophotometer (TU 1901, Puxi, Beijing, China). With the help of an exciting wavelength of 300 nm, photoluminescence (PL) spectra were gathered on a fluorescence spectrophotometer (LS55, PE, Waltham, MA, USA).

### 2.4. Photoelectrochemical Measurements

The spectra of photocurrent and electrochemical impedance were recorded on a electrochemical workstation (CHI660E, Chenhua Instruments Co., Shanghai, China) equipped with a traditional standard three-electrode system including the saturated Ag/AgCl reference electrode, the Pt wire counter electrode and the as-prepared photocatalysts working electrode. Such measurement details were already reported in our previous research [42].

### 2.5. Photocatalytic Experiments

Photodegradation experiments of MO solution (4.0 × 10^−5^ mol L^−1^) were accomplished in a homemade equipment, as is seen in Figure S1. A similar photodegradation procedure for RhB solution was previously reported in our recent study [43,44,45]. Put slightly differently, 60 mL MO aqueous solution (4.0 × 10^−5^ mol L^−1^) or 50 mL TCH aqueous solution (10 mg L^−1^) was used in each test, and the dosage of photocatalysts was equally 100 mg. Under a given time of visible-light irradiation, the concentration of the resulting solution was determined with the aid of a UV–vis spectrophotometer recording variable intensities of the characteristic absorbance peak (464 nm for MO, and 357 nm for TCH).

## 3. Results and Discussion

### 3.1. Phase Structure and Composition

**Figure 1:**
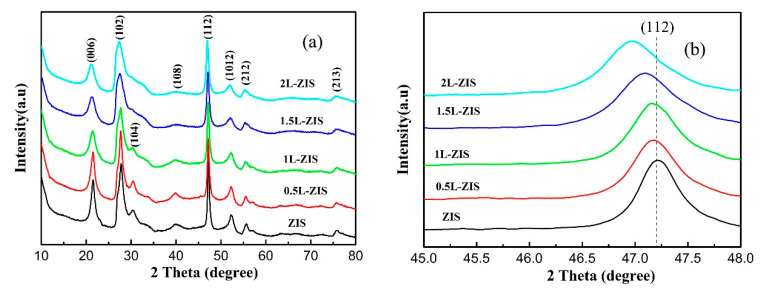
(**a**) X–ray diffractometer (XRD) patterns of Pure ZnIn_2_S_4_ (P-ZIS) and doped ZnIn_2_S_4_ (ZIS) samples; (**b**) partially enlarged diffraction peak of (112) plane of P-ZIS and doped ZIS samples.

**Figure 2:**
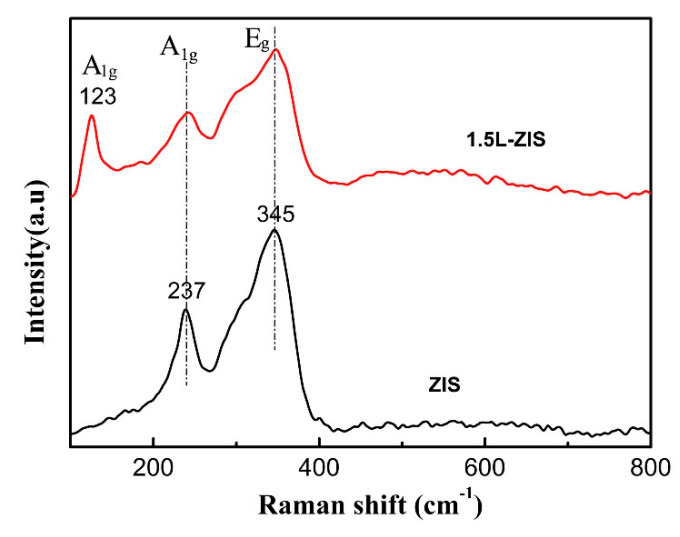
Raman spectra of P-ZIS and 1.5 at%-doped ZnIn_2_S_4_ (1.5L-ZIS) samples.

**Figure 3:**
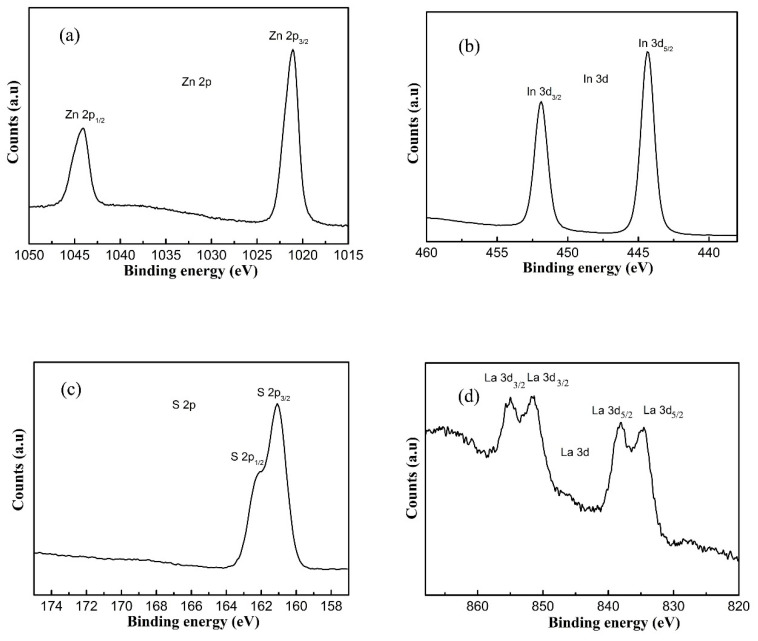
High resolution X–ray photoelectron spectroscopy (XPS) spectra of 1.5L-ZIS sample: (**a**) Zn 2p; (**b**) In 3d; (**c**) S 2p; (**d**) La 3d.

The X–ray diffractometer (XRD)patterns of P-ZIS and La-doped ZIS are presented in Figure 1a, from which it can draw a conclusion that all the diffraction peaks of pristine ZIS coincide perfectly with those of hexagonal ZnIn_2_S_4_ (JCPDS No. 65-2056). Specifically, eight notable diffraction peaks located at about 2*θ* angles of 21.6°, 27.8°, 30.4°, 40°, 47.2°, 52.1°, 56.1°, and 75.9° are respectively indexed to be (006), (102), (104), (108), (112), (1012), (202), and (213) planes of ZIS, agreeing well with previous studies [7,9,13,31]. As for doped ZIS samples, an obvious change occurred to the diffraction peaks, perhaps due to the introduction of La^3+^ into ZIS crystals. First, the intensities of (104) and (108) planes of doped ZIS samples gradually decreased with increasing the concentration of La dopant. Secondly, as is seen in Figure 1b, the phenomenon that the representative diffraction peak of (112) crystal plane of doped ZIS samples slightly shifted to left compared with that of P-ZIS is observable, implying that La ions have incorporated into the crystal lattice of ZIS and enlarged the crystal plane. This shift might be ascribed to the fact that the radius of La ion (1.06 nm) is larger than that of In ion (0.81 nm). Additionally, the diffraction peak of (102) crystal plane of doped ZIS samples also shifted left, which was similar to that of the (112) crystal plane. In order to confirm the existence of La ions, Raman spectra of P-ZIS and 1.5L-ZIS samples are displayed in Figure 2. According to previous report [46], two prominent peaks centered at about 237 cm^−1^ (A_1g_ mode) and 345 cm^−1^ (E_g_ mode) in two curves are probably related to the vibration in Me-S (Me = Zn, In) tetrahedral sites and symmetric stretching of the S–S bonds in the octahedral structure, respectively. As for the 1.5L-ZIS sample, there appeared a well-defined peak at about 123 cm^−1^, matching with the previously reported result of La_2_S_3_ Raman spectrum [47]. XPS were utilized to further verify the existence and reveal the chemical valence status of La ions in ZnIn_2_S_4_ crystal lattice. From Figure 3, four surface elements of Zn, In, S, and La can be detected in the 1.5L-ZIS sample. Apparently two sharp peaks with binding energies of 1021.1 eV and 1044.1 eV are respectively resulted from Zn 2p_3/2_ and Zn 2p_1/2_ (Figure 3a). As can be noted from the high-resolution XPS spectrum of In 3d (Figure 3b), two characteristic peaks located at 451.9 eV and 444.4 eV are closely related to In 3d_5/2_ and In 3d_3/2_ valence state. The S 2p spectrum exhibits two peaks with binding energies of 161.1 eV and 162.1 eV in Figure 3c, which is well matched with S^2−^ in ZIS. As for La 3d peaks (Figure 3d), there appears four peaks with binding energies of 834.5 eV, 838.1 eV, 851.5 eV and 855.1 eV which can be assigned to La^3+^ of La_2_S_3_ (as seen in the Supplementary Materials). Based above, the doping of La^3+^ ions into ZIS can be successfully ascertained by the aid of XPS results combining with XRD patterns and Raman spectra.

### 3.2. Morphology and Brunauer-Emmett-Teller (BET) Analysis

**Figure 4:**
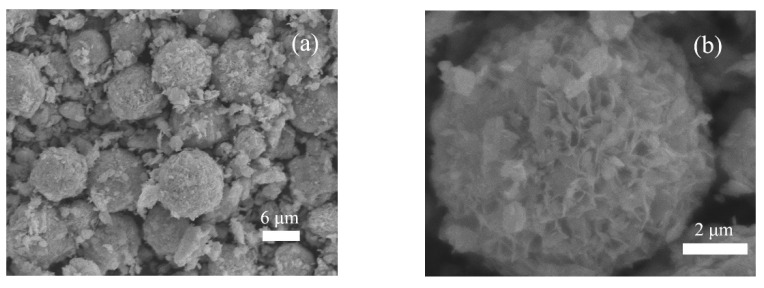
(**a**) Low magnification scanning electron microscope (SEM) image; (**b**) high magnification SEM image of the 1.5L-ZIS sample.

**Figure 5:**
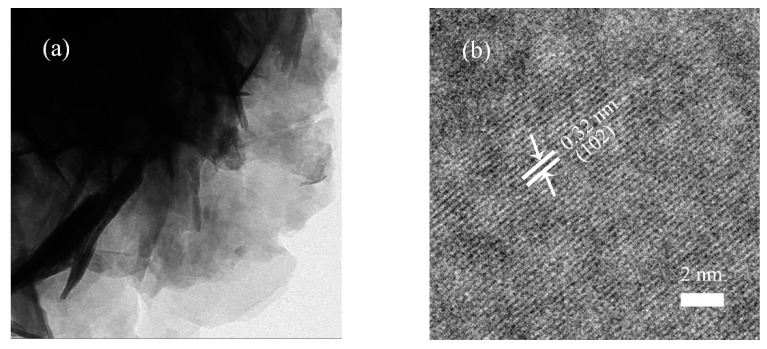
(**a**) Transmission electron microscopy (TEM) image; (**b**) high resolution transmission electron microscopy (HRTEM) image of the 1.5L-ZIS sample.

Figure 4 shows the scanning electron microscope (SEM) images of the 1.5L-ZIS sample. As is seen from low-magnification SEM image (Figure 4a), a substantial amount of crystallites were self-organized into flower-like microspheres with inhomogeneous diameters ranging from 2 μm to 10 μm after the hydrothermal treatment process. From the magnified SEM image (Figure 4b), these floriated microspheres possessed a special porous structure, in which numerous petal-like distorted nanosheets were arranged and intertwined at random. Such a unique feature would provide much larger surface area and more active sites for photodegradation reaction. The detailed microstructure of the 1.5L-ZIS sample was further investigated by transmission electron microscope (TEM) and high resolution transmission electron microscope (HRTEM). As shown in Figure 5a, we can find some curved and stacked nanosheets from the side view of a single microsphere. From Figure 5b, we can consider that the adjacent lattice fringe with 0.32 nm is associated with the (112) crystallographic plane of ZIS according to previous studies [9]. Additionally, a slight lattice deformation was clearly seen as well, possibly caused by the incorporation of La^3+^ into ZIS lattice.

**Figure 6:**
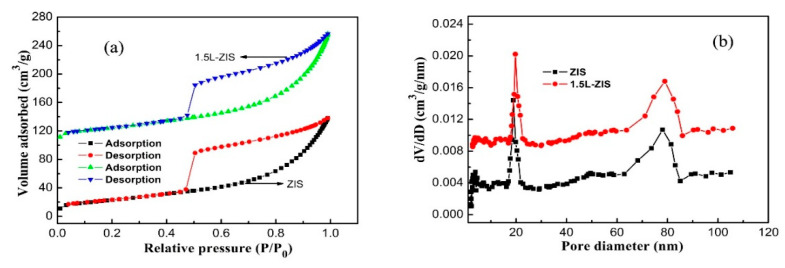
(**a**) Nitrogen adsorption–desorption isotherm; (**b**) corresponding pore size and distribution of the 1.5L-ZIS sample.

The nitrogen adsorption-desorption isotherms of P-ZIS and 1.5L-ZIS samples are portrayed in Figure 6. On the whole, the adsorption-desorption isotherm of 1.5L-ZIS sample is very similar to that of P-ZIS. Each of two samples presented a type IV curve combined with an obvious H_3_-type hysteresis loop according to the International Union of Pure and Applied Chemistry (IUPAC) classification, from which the characteristic of mesoporous structure can be rationally inferred. From Figure 6b, we can find that the corresponding pore distribution of the two samples is relatively wide, ranging from about 2 nm to 100 nm. Not only does it authenticate the inference of the existence of mesopores, but also it is well indicative of the formation of macropores. Additionally, the Brunauer-Emmett-Teller (BET) surface areas of P-ZIS and 1.5L-ZIS could be individually estimated to be about 84.2 and 90.5 m^2^/g according to the results of adsorption-desorption isotherms, meaning that a slight increase in Brunauer-Emmett-Teller (BET) surface area of 1.5L-ZIS was obtained after the incorporation of La^3+^ into the ZIS crystal lattice. As mentioned above, the 1.5L-ZIS sample will be endowed with good absorption capacity due to its larger BET specific surface area.

### 3.3. Optical Characterization

**Figure 7:**
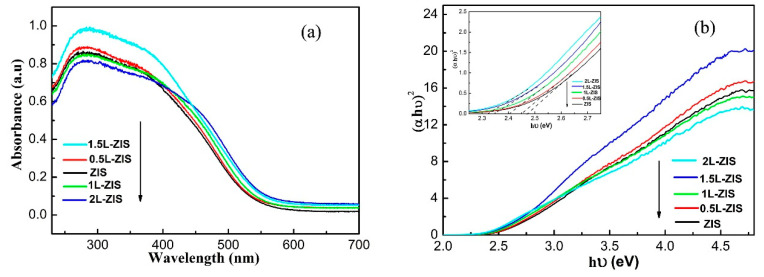
(**a**) Ultraviolet–visible (UV–vis) absorbance spectra of P-ZIS and La-doped ZIS samples; (**b**) the derived plots of (*αhv*)^2^ versus *hv* from the absorption spectrum for P-ZIS and La-doped ZIS samples with partially magnified absorption spectra inset.

It is believed that the optical absorption characteristic of one photocatalyst is intimately associated with its electronic structure and energy band structure. Consequently, different photocatalysts display different absorption features, either in the light absorbance intensity or light absorbance range. Thus, the light absorption characteristics of P-ZIS and doped ZIS were investigated by UV-vis diffuse reflection spectroscopy (DRS) to evaluate the effect of La doping on the optical property. As is shown in Figure 7a, P-ZIS displayed a strong absorption edge at around 560 nm, demonstrating its good visible-light absorption capacity. After the incorporation of La^3+^ ions into ZIS lattice, a slight red-shift could be easily found in the adsorption band of doped ZIS, suggesting that the response range of visible light is widened and the optical absorption ability is enhanced to some extent because of the narrowed band gap. More specifically, a continuous red-shift toward the long wavelength direction could also be found in the adsorption band of doped ZIS with the increase of La doping content from 0 to 2at%. According to a previous study, the extrapolation method was employed to quantify the variation of band gap energy of doped ZIS based on the Kubelka–Munk equation. To serve this purpose, the plots of *hν* with respect to (*αhν*)^2^ for P-ZIS and doped ZIS were presented to estimate band gap energies. Through extrapolating the linear portion of above plots, the band gap energies of P-ZIS, 0.5L-ZIS, 1L-ZIS, 1.5L-ZIS, and 2L-ZIS samples were individually determined to be about 2.48 eV, 2.45 eV, 2.4 eV, 2.35 eV, and 2.32 eV, as is shown in the inset of Figure 7b. The above results verified that the band gap of doped ZIS samples indeed was narrowed by the incorporation of La ions, which was consistent with the results of XRD, Raman and XPS spectra.

### 3.4. Photocatalytic Activities

**Figure 8:**
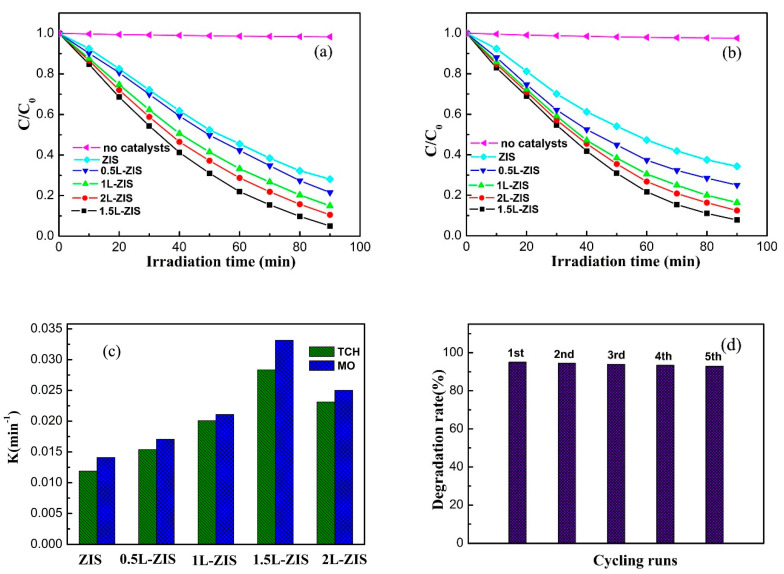
(**a**) The photodegradation performance of methyl orange (MO) solution; (**b**) the photodegradation performance of tetracycline hydrochloride (TCH) solution; (**c**) the corresponding apparent reaction rate constant; (**d**) cycling performance of the photodegradation of MO solution over the 1.5L-ZIS sample.

MO dye is one of the most widely used pollutants in the photodegradation experiments. In our work, we first took MO dye as model pollutant to investigate the photocatalytic efficiency of P-ZIS and doped ZIS photocatalysts. Figure 8a presents the photodegradation curves of MO over different photocatalysts. The concentration of MO exhibited little change in the absence of catalysts under visible-light irradiation. The concentration of MO obviously decreased over photocatalysts with irradiation time prolonging, as shown in Figure 8a. This phenomenon indicated that MO was able to be photodegraded by P-ZIS, although its photodegradation rate was relatively low. Interestingly, the incorporation of La^3+^ ions made a great contribution to the improvement of the photodegradation rate of MO. Specifically, the photodegradation rate increased with the increase of the doping content; when the concentration of La was lower than 1.5 at%. After that, a further increase of La doping content resulted in a decrease of the photodegradation rate of MO. From Figure 8a, 1.5L-ZIS had the highest photocatalytic efficiency among all the tested samples, and its photodegradation rate was about 95% when the irradiation time reached 90 min. Simultaneously, we can find that the color of the suspension continually became lighter with prolonging irradiation time, as is shown in Figure S2. In our work, the optimal loading percent of La was 1.5 at%. If the loading percent is more than the critical value (1.5 at%), La doping would give rise to the formation of excess recombination centers, which resulted in the decrease of photodegradation efficiency.

As a category of wide-spectrum bacteriostatic drugs with bactericidal effect, TCH has a priority in the choice of treatment for non-bacterial infections including chlamydia infection, rickets disease, mycoplasma pneumonia and relapses fever. Notably, TCH antibiotics are in an active form which can be transferred into the environment, especially waste water, via excretion from human beings and animals after medication. Such a result will pose serious threats to human health and ecological equilibrium because of the common development of multiresistant chains of microorganisms [48,49]. Thus, it is an essential and critical issue to remove TCH antibiotics from polluted water. Based on the above, we carried out TCH photodegradation tests over P-ZIS and doped ZIS photocatalysts. Figure 8b shows the variation of TCH concentration with respect to irradiation time over different photocatalysts. The variation of the concentration of TCH was similar to that of MO in the absence of catalysts due to the excellent stability. Furthermore, the result of TCH photodegradation was also analogous to that of MO photodegradation. Specifically, the photodegradation rate of TCH first increased with the increase of La doping concentration, then decreased subsequently when the La concentration exceeded 1.5 at%. Interestingly, the highest photodegradation rate (92%) for TCH was obtained after a constant irradiation time of 90 min.

Previous studies have found that the photodegradation reaction of MO and TCH followed the pseudo-first order kinetics. Herein, the corresponding kinetic constant (*k*) values for MO and TCH photodegradation are presented in Figure 8c. As we can observe, either for MO photodegradation or TCH photodegradation, the effect of La doping content on the kinetic constant *k* was identical to that of the photodegradation rate. The maximum k value for MO or TCH photodegradation reached up to 0.0283 min^−1^ or 0.0331 min^−1^ respectively when the La dosage was 1.5 at%. Additionally, each *k* value for MO photodegradation was slightly larger than that for TCH photodegradation over the identical photocatalyst. The results of photocatalytic activities over 1.5L-ZIS not only demonstrated effective photodegradation of MO and TCH pollutant, but also verified the selectivity for photodegradation. Since the stability should be seriously considered in practical applications, the enduring stability of the 1.5L-ZIS photocatalyst was examined by recycling the photocatalytic oxidation of MO for five times under the identical conditions. As displayed in Figure 8d, only a slight loss of photodegradation rate for MO was found, indicating the high efficiency, excellent stability and recyclability in potential practical application for the treatment of water contamination.

### 3.5. Possible Photocatalytic Mechanism

**Figure 9:**
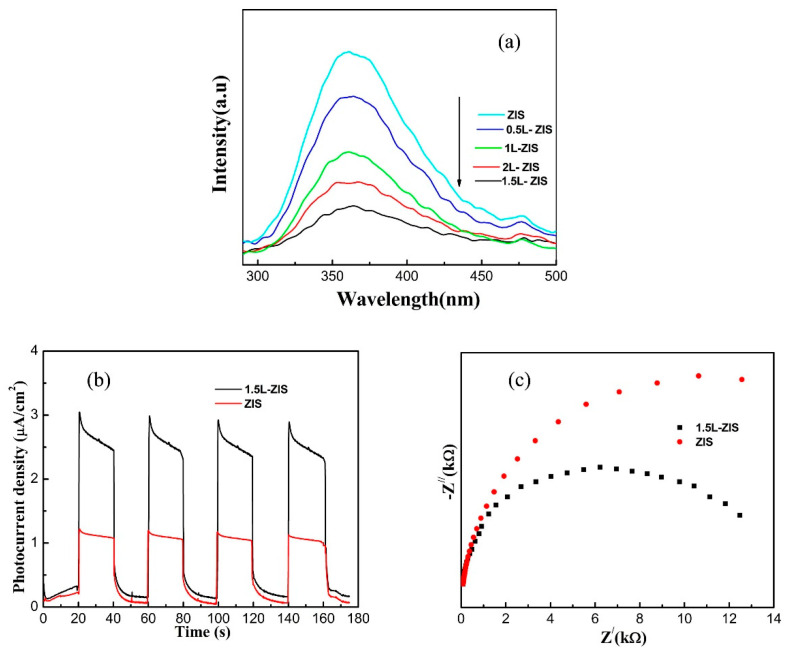
(**a**) Photoluminescence (PL) spectra of P-ZIS and doped ZIS samples; (**b**) transient photocurrents; (**c**) electrochemical impedance spectra of pristine ZIS and 1.5L-ZIS electrodes under visible light irradiation.

As demonstrated above, the difference in morphology and BET surface area was negligible between P-ZIS and 1.5L-ZIS samples. Thus, we can infer that the remarkable improvement of photodegradation rate of MO and TCH was most likely to be irrelevant to the two factors. From the results of DRS, there is no doubt that the enhancement of light absorption ability must make a positive contribution to the improvement of the photodegradation rate. Besides the above, the factor that La doping regulated the electronic structure for doped ZIS photocatalyst was probably crucial to the separation and transfer of photo-activated electron-holes. To validate the hypothesis, PL spectra, photocurrent and electrochemical impedance spectroscopy (EIS) curves are respectively presented in Figure 9a. Among all tested samples, we can find that P-ZIS owned the highest emission intensity, indicating its high recombination rate of photoactivated charge carriers. After La doping, the emission intensity of the PL spectrum of doped ZIS samples continuously decreased until the concentration of La ions was up to 1.5 at%. Obviously, the 1.5 L-ZIS sample had the weakest emission intensity, revealing that it possessed the lowest recombination rate of photoactivated charge carriers among all tested samples. That is to say that efficient separation of photoactivated charge carriers was completely accomplished in the 1.5L-ZIS sample. Next, the charge transfer could further be investigated by photocurrent-time and EIS experiments. Figure 9b presents the photocurrent-time curves of P-ZIS and 1.5L-ZIS samples with four intermittent visible-light irradiation cycles, from which we could notice that the photocurrent of the 1.5L-ZIS sample was almost two times larger than that of P-ZIS, meaning its higher separation and transfer rate of photo-activated charge carriers. Generally, the larger the arc radius, the lower the separation rate of photo-activated charge carriers. It is clear from Figure 9c that the arc radius of the 1.5L-ZIS sample is much smaller than that of P-ZIS, being indicative of its faster interfacial charge transfer and more efficient separation along the interface. Thus, the above discussion was favorable to the role that La doping played in suppressing the recombination rate of photoactivated charge carriers and elevating the rate of interfacial charge separation.

**Figure 10:**
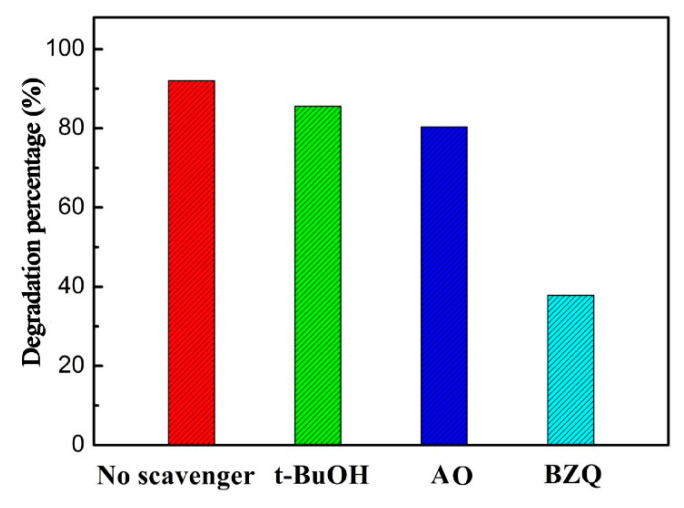
Effect of various scavengers on the visible light photocatalytic performance of 1.5L-ZIS sample toward the photodegradation of TCH.

In order to examine in depth the photodegradation mechanism of TCH over 1.5L-ZIS photocatalyst, we designed radical trapping experiments, in which benzoquinone (BZQ 4 mmol L^−1^), tert-butyl-alcohol (*t*-BuOH 4 mmol L^−1^), and ammonium oxalate (AO 4 mmol L^−1^) were determined to act as scavengers for capturing, ·O_2_^−^, ·OH, and *h*^+^, respectively. As seen in Figure 10, the photodegradation rate of TCH decreased a little after the introduction of AO or *t*-BuOH, demonstrating that ·OH or *h*^+^ posed a limited effect on the photodegradation reaction of TCH. However, the addition of BZQ significantly suppressed the photodegradation reaction of TCH, suggesting that ·O_2_^−^ was the critical radical which overwhelmingly dominated the photodegadation rate of TCH in the 1.5L-ZIS photocatalyst system.

**Figure 11:**
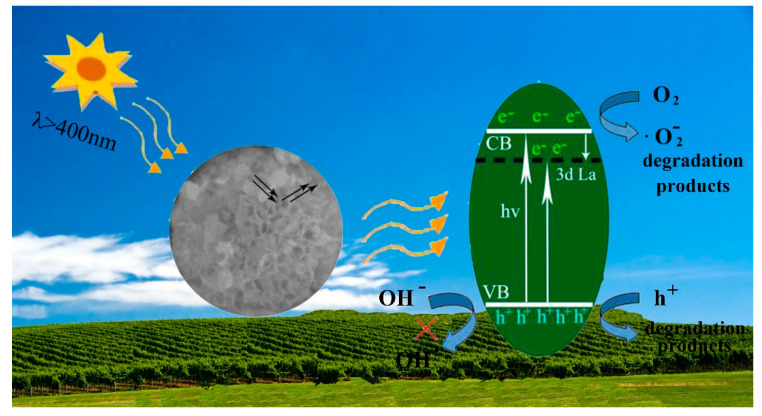
Schematic diagram of proposed reaction mechanism over La-doped ZIS system under visible-light irradiation.

According to previous studies [50], the conduction band (CB) potential of a single semiconductor can be speculated about in theory by using the empirical equation of *E*_CB_ = *X* − *E*_e_ − 0.5*E*_g_, where *E*_CB_ represents CB edge potential, *X* is the absolute electronegativity, *E*_g_ and *E*_e_ respectively refer to the band gap energy and energy of free electrons on the hydrogen scale (~4.5 eV). Here, the *X* value is about 4.894 eV [51]. In combination with the results of DRS, the CB potential of ZIS was calculated to be about −0.806 eV. Subsequently, the valence band (VB) potential of ZIS was determined to be 1.674 eV by the aid of the equation of *E*_VB_ = *E*_CB_ + *E*_g_. Based on the above, a possible schematic mechanism for the significant enhancement of photodegradation behavior is presented in Figure 11. Under visible-light irradiation, the electrons from VB position of ZIS are easily activated, then transfer to the CB position due to its smaller band gap. As for La-doped ZIS samples, In^3+^ is substituted by La^3+^ into the crystal lattice. Accordingly, a shallow energy level will be formed below the CB position of ZIS. The shallow energy level formed by La^3+^ doping can not only promote light absorption ability and generate more electron-hole pairs, but also can trap the photogenerated electrons from the CB surface and facilitate the effective separation of photogenerated charge carriers. Actually, the accumulated electrons on the CB of ZIS (*E*_CB_ = −0.806 eV) are negative enough to be capable of reducing O_2_ to generate the product of ·O_2_^−^ (*E*^0^ (O_2_/·O_2_^−^) −0.33 eV vs. Normal Hydrogen Electrode (NHE)) [52,53], affording more active species to be involved in the photodegradation reaction. Although the remaining holes on VB of ZIS (1.46 eV vs. NHE) is not oxidative enough to make OH^−^ transform into ·OH active species (*E*^0^ OH^−^/·OH (1.99 eV) vs. NHE) [54], they could straightforwardly oxidize organic molecular into CO_2_ and H_2_O. Such a result was in good concordance with that of the trapping experiment. Based on the above, a significant enhancement of photodegradation performance was successfully achieved in the La-doped ZIS photocatalyst system through regulating the electronic structure and facilitating the effective separation of photo-activated charge carriers.

## 4. Conclusions

In summary, La-doped ZnIn_2_S_4_ microspheres were achieved in a facile way via a one-pot hydrothermal synthesis route. The experimental results verified that La doping exerted a substantial effect on optical properties and photodegradation behavior. After the incorporation of La ions, the light absorption capacity was obviously strengthened, and more efficient separation of photo-activated charge carriers was indeed accomplished due to the formation of the doping energy level. Compared with P-ZIS, the resultant 1.5L-ZIS displayed a great enhancement in the photodegradation of MO and TCH. It is believed that the synergistic effect of the elevated visible-light absorption capacity and efficient photo-activated charge carriers’ separation mainly contributes to the great improvement of photodegradation behavior. Our work will pave the way to designing a novel and simple synthetic route for the preparation of high-efficiency visible light-responsive photocatalysts.

## Supplementary Materials

The following are available online at https://www.mdpi.com/2079-4991/10/10/2026/s1: Figure S1: Schematic image of the setup for photodegradation experiments, Figure S2: The variation of color (methyl orange (MO)) during the photodegradation reaction over 1.5L-ZIS (1.5 at%-doped ZnIn_2_S_4_) catalyst.
