# Supplementary material for: One-Pot Hydrothermal Synthesis of La-Doped ZnIn2S4 Microspheres with Improved Visible-Light Photocatalytic Performance
Source: Nanomaterials (Basel). 2020 Oct 14;10(10):2026. doi: 10.3390/nano10102026 (PMC7602468; doi:10.3390/nano10102026)
Supplement: Supplementary file 1 [file nanomaterials-10-02026-s001.pdf]

nanomaterials

Supplementary Materials

One-Pot Hydrothermal Synthesis of La-Doped $ZnIn_2S_4$ Microspheres with Improved Visible-Light Photocatalytic Performance

Tiekun Jia [1,*], Ming Liu [1,*], Chunyang Zheng [1], Fei Long [2], Zhiyu Min [1,*], Fang Fu [1], Dongsheng Yu [1], Jili Li [1], Joong Hee Lee [3] and Nam Hoon Kim [3]

[1] School of Materials Science and Engineering, Luoyang Institute of Science and Technology, Luoyang 471023, China; cocoaa99@163.com (C.Z.); fufang1@126.com (F.F.); dongsh_yu@163.com (D.Y.); lijili328@126.com (J.L.)

[2] School of Materials Science and Engineering, Guilin University of Technology, Guilin 541004, China; longf@glut.edu.cn

[3] Department of Nano Convergence Engineering, Jeonbuk National University, Jeonju 54896, Korea; jhl@jbnu.ac.kr (J.H.L.); nhk@jbnu.ac.kr (N.H.K.)

* Correspondence: tiekunjia@126.com or tiekun_jia@lit.edu.cn (T.J.); liumingming4455@163.com (M.L.); mindayu@163.com (Z.M.); Tel./Fax: +86-(37)-965928196 (T.J.)

1. Experimental Section

Photocatalytic Experiments

Photodegradation experiments were carried out using homemade equipment, as seen in Figure S1. Three daylight lamps (30 W for each, $\lambda \geq 400$ nm) were designated as the visible-light source for triggering the photodegradation reaction. The dish filled with methyl orange (MO) or tetracycline hydrochloride (TCH) solution was placed under the daylight lamp, and the distance between the dish and the daylight lamp was about 10 cm. The light intensity was about 450 mW/cm^2. In a typical run, a thin layer of film (100 mg photocatalyst) was dispersed into the dish containing MO (60 mL, 4.0×10^{-5} mol L^{-1}) or TCH (60 mL, 10 mg L^{-1}) aqueous solution. Prior to irradiation, the obtained suspension was stirred in the dark for 1 h to reach an adsorption-desorption equilibrium among the catalyst, MO (or TCH) and water. At given irradiating time intervals, 6 mL reacted solution was centrifuged to measure the concentration variation of MO (or TCH) solution by recording the variation of the intensity of absorption peak (464 nm for MO, and 357 nm for TCH) using an ultraviolet (UV)-visible spectrophotometer (TU1901, Puxi, China). The photodegradation of efficiency of MO (or TCH) under visible light irradiation was expressed by C/C_0, where C is concentration of the MO (or TCH) solution at a certain irradiation time t, C_0 is the initial concentration of MO (or TCH) solution after adsorption-desorption equilibrium.

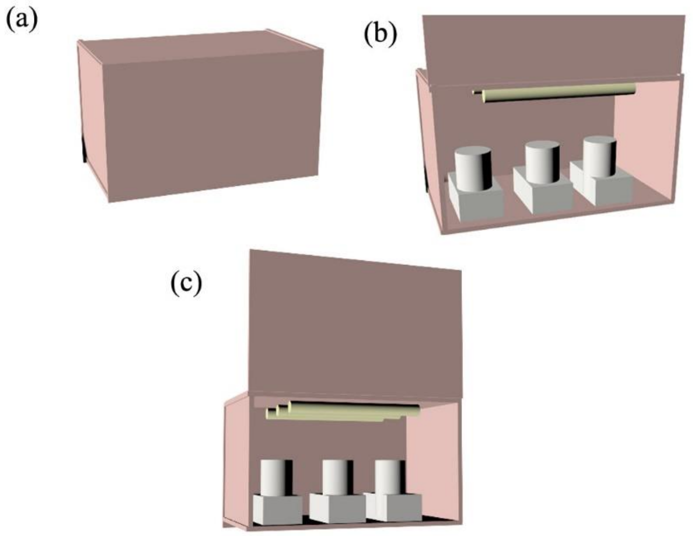

Figure S1. Schematic image of the setup for photodegradation experiments.

2. Phase Structure and Composition

In our experiment, the tiny amount of La is mostly likely to form the phase of La_2S_3. The explanation is described as follows. In our work, no starting materials containing alkali (OH^-) or carbonate were introduced into the reaction system except a sulfur source. According to previous studies [1,2], other lanthanide-based compounds, such as $La(OH)_3$ and $La_2O_2CO_3$, could be formed in the presence of NaOH or $(NH_2)_2CO$. That is to say, no condition is satisfied to form $La(OH)_3$ or $La_2O_2CO_3$. On the other hand, it has been proved that some dopant ions, such as La^{3+}, Ce^{3+}, Gd^{3+}, Er^{3+} or Y^{3+}, can be successfully introduced into $ZnIn_2S_4$ [3,4]. Furthermore, no O1s X–ray photoelectron spectroscopy (XPS) peak was detected in the doped $ZnIn_2S_4$ sample. Based on above, it can be rationally inferred that La ions are mostly likely to substitute In ions to form the phase of La_2S_3.

3. Photocatalytic Performance

The color of the suspension continually became lighter with prolonging irradiation time, as shown in Figure S2.

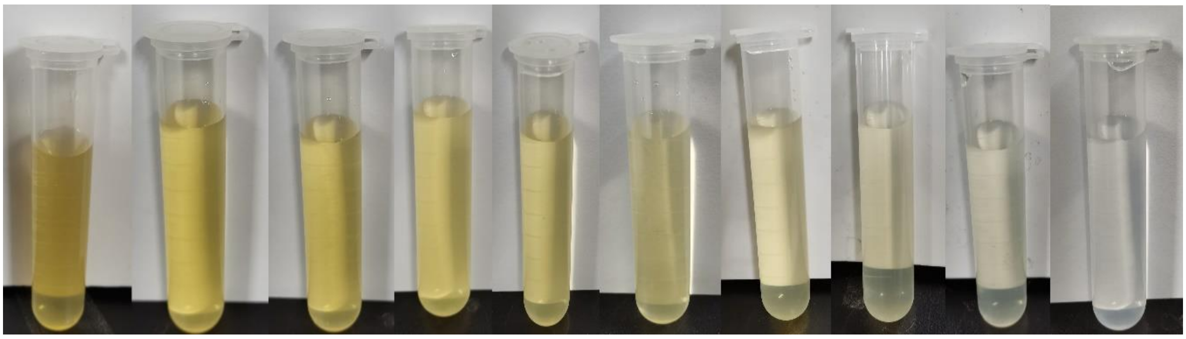

Figure S2. The variation of color (methyl orange (MO)) during the photodegradation reaction over 1.5L-ZIS (1.5 at%-doped $ZnIn_2S_4$) catalyst.

References

1. Yadav, A.A.; Lokhande A.C.; Lokhande C.D. A simple chemical route for synthesis of microrods-like La_2O_3 thin films. *Mater. Lett.* **2015**, 160, 500–502.
2. Chen, L.; Li, Y.; Sun, Y.; Chen, Y.; Qian J. $La(OH)_3$ loaded magnetic mesoporous nanospheres with highly efficientphosphate removal properties and superior pH stability. *Chem. Eng. J.* **2019**, *306*, 342–348.

3. Tian, F.; Zhu, R.; He, Y.; Ouyang, F. Improving photocatalytic activity for hydrogen evolution over $ZnIn_2S_4$ under visible-light: a case study of rare earth modification. *Int. J. Hydrogen Energy* **2014**, *39*, 6335–6344.
4. Zhu, R.; Tian, F.; Che S.; Cao, G.; Ouyang, F. The photocatalytic performance of modified $ZnIn_2S_4$ with grapheneand La for hydrogen generation under visible light. *Renewable Energy*, **2017**, 113, 1503–1514.

Publisher's Note: MDPI stays neutral with regard to jurisdictional claims in published maps and institutional affiliations.

© 2020 by the authors. Submitted for possible open access publication under the terms and conditions of the Creative Commons Attribution (CC BY) license (http://creativecommons.org/licenses/by/4.0/).
